# Supplementary material for: In-House Implementation of Tumor Mutational Burden Testing to Predict Durable Clinical Benefit in Non-Small Cell Lung Cancer and Melanoma Patients
Source: Cancers (Basel). 2019 Aug 29;11(9):1271. doi: 10.3390/cancers11091271 (PMC6769455; doi:10.3390/cancers11091271)
Supplement: Supplementary file 1 [file cancers-11-01271-s001.zip › cancers-564639-suppl-final.docx]

Supplementary Materials: In-house Implementation of Tumor Mutational Burden in Real World to Predict Durable Clinical Benefit in Non-Small Cell Lung Cancer and Melanoma Patients

Simon Heeke, Jonathan Benzaquen, Elodie Long-Mira, Benoit Audelan, Virginie Lespinet, Olivier Bordone, Salomé Lalvée, Katia Zahaf, Michel Poudenx, Olivier Humbert, Henri Montaudie, Pierre-Michel Dugourd, Madleen Chassang, Thierry Passeron, Hervé Delingette, Charles-Hugo Marquette, Véronique Hofman, Albrecht Stenzinger, Marius Ilié and Paul Hofman

[Methods S1: Sample selection and preparation. 2](#_Toc17878407)

[Methods S2: DNA extraction and sequencing. 2](#_Toc17878408)

[Methods S3: Bioinformatics and data analysis. 3](#_Toc17878409)

[Methods S4: Immunohistochemistry for PD-L1 and CD8 expression. 3](#_Toc17878410)

[Methods S5: Machine learning and mathematical modeling. 3](#_Toc17878411)

[Figure S1: Flowchart of samples tested using the different sequencing panels. 4](#_Toc17878412)

[Figure S2: Oncoprint of genomic and clinical features of the NSCLC cohort as tested by FoundationOne^®^. 5](#_Toc17878413)

[Figure S3: Oncoprint of genomic and clinical features of the melanoma cohort as tested by FoundationOne^®^. 6](#_Toc17878414)

[Figure S4: Turn-around time for samples sent to Foundation Medicine. 7](#_Toc17878415)

[Figure S5: Deamination score of samples sequenced using the Oncomine™ TML panel. 8](#_Toc17878416)

[Figure S6: Impact of different deamination protocols on the C to T conversion rate (C > T) and the TMB using the Oncomine™ TML panel. 9](#_Toc17878417)

[Figure S7: Influence of tumor cell content on sequencing results. 10](#_Toc17878418)

[Figure S8: Comparison of sequencing results between FoundationOne^®^ and Hotspot V2. 11](#_Toc17878419)

[Figure S9: Receiver operator characteristics (ROC) curves calculated for the assessment of DCB in the NSCLC cohort. 12](#_Toc17878420)

[Figure S10: Correlation of PD-L1 expression in tumor cells and TMB with clinical outcome. 13](#_Toc17878421)

[Figure S11: Logistic regression models built to predict durable clinical benefit (DCB) in the NSCLC cohort. 14](#_Toc17878422)

[Figure S12: Packyears of smoking as biomarker. 15](#_Toc17878423)

[Table S1: Characteristics of non-small cell lung cancer patients. 16](#_Toc17878424)

[Table S2: Characteristics of melanoma patients. 17](#_Toc17878425)

[Table S3: Prediction of DCB from a single biomarker in non-small cell lung cancer patients. 18](#_Toc17878426)

[Table S4: Prediction of DCB from a single biomarker in melanoma patients. 18](#_Toc17878427)

[Table S5: Prediction of durable clinical benefit in non-small cell lung cancer using single biomarkers. 19](#_Toc17878428)

[Table S6: Prediction of durable clinical benefit in non-small cell lung cancer patients by combining two biomarkers using decision trees (*N* = 24). 19](#_Toc17878429)

[Table S7: Prediction of durable clinical benefit in non-small cell lung cancer patients by combining two biomarkers using logistic regression models (*N* = 24). 20](#_Toc17878430)

[Table S8: Integrating packyears of smoking as biomarker for the prediction of DCB in non-small cell lung cancer patients using logistic regression models (*N* = 24). 20](#_Toc17878431)

[Table S9: Prediction of durable clinical benefit in melanoma patients using single biomarkers. 21](#_Toc17878432)

[Table S10: Prediction of durable clinical benefit in melanoma patients by combining two biomarkers using decision trees (*N* = 22). 21](#_Toc17878433)

[Table S11: Prediction of durable clinical benefit in melanoma patients by combining two biomarkers using logistic regression models (*N* = 22). 22](#_Toc17878434)

Table S12 see the Excel table

Supplementary Methods 1: Sample Selection and Preparation

Formalin-fixed and paraffin embedded (FFPE) tumor specimens from various clinical sources were collected from lung adenocarcinoma patients at the Laboratory of Clinical and Experimental Pathology (LPCE; University Hospital of Nice, CHU of Nice, France). All patients had a clinical indication for molecular testing and were informed about the purpose of the molecular analysis by the treating physician. All samples were processed by the routine diagnostic pipeline of the LPCE between 2016 and 2018. A total of 96 samples were selected.

Samples were required to have a tumor size of at least 25 mm² (according to the sample instructions for FoundationOne) and to allow the cutting of >20 consecutive 5 µm sections to have sufficient material for all pre-planned analysis (10 unstained sections for FoundationOne^®^ testing, one section for Hematoxylin & Eosin staining, four sections for the extraction of DNA for in-house sequencing, and two slides for the immunohistochemistry analyses of CD8 and PD-L1). Samples with a tumor content of at least 20% were considered for the analysis.

For genetic testing using FoundationOne^®^, 10 unstained sections of 5 µm each were prepared and sent to Foundation Medicine (Cambridge, MA, USA) using the provided FoundationOne^®^ testing kit. Additionally, one Hematoxylin and Eosin pre-stained tumor section was sent to Foundation Medicine.

Supplementary Methods 2: DNA Extraction and Sequencing

For DNA isolation, four tumor sections of 5 µm each were cut on a microtome. The DNA isolation was carried out using the Maxwell® 16 Tissue DNA Purification Kit (ref AS1030 Promega, Madison, WI, USA). Nucleic acid concentration was measured with the Qubit dsDNA HS assay kits on the Qubit 2.0 Fluorometer (Q32851, Thermo Fisher Scientific, Waltham, MA, USA).

Hotspot v2 Sequencing

A total of 10 ng of DNA was processed using the Ion AmpliSeq™ Cancer Hotspot Panel v2 Panel (ref 4475346) using the Ion AmpliSeq™ Library Kit™ (ref 4480441; Ion PGM™, Thermo Fisher Scientific). Samples were barcoded and libraries from each sample were pooled at a 25 pM concentration. Library quality and quantity was assessed using the Agilent High Sensitivity DNA Kit (ref 5067462, Agilent Technologies, Santa Clara, CA, USA). Ion chef was used for automated template preparation (A34461), enrichment of ion spheres and chip loading on an Ion 510 Chip (ref A34292) for sequencing using the Ion S5™ System (Thermo Fisher Scientific). Results were analyzed using the variant caller by aligning the reads to the hg19 reference genome, calling the variants, and generating an interactive report for visualization and quality control.

Oncomine™ TML Sequencing

The Oncomine™ Tumor Mutation Load Assay is a PCR-based next-generation sequencing assay (Oncomine™ Mutation Load Research Assay, ref A37908, TML; Thermo Fisher Scientific,) which covers 409 genes spanning 1.65 megabases (Mb).

The libraries were prepared using the Ion Chef System (A37910 and A30011) and 10 ng of DNA was used for each of the two primer pools (20 ng of DNA per sample in total). Eight samples were multiplexed on an Ion 540 chip per run. Sequencing was performed on an Ion S5 benchtop sequencer and reads were aligned to hg19 using Torrent Suite 5.6. Generated BAM files that were transferred to Ion Reporter 5.10 for variant calling and secondary analysis including TMB calculation. The assay is labeled as research use only and not certified for diagnostic procedures in the European Union.

Deamination Repair

Where indicated, deamination repair was performed using a heat-labile Uracil-DNA-Glycosylase (UDG; Thermo Fisher Scientific, ref 78310100UN). After DNA isolation and directly prior to library preparation, DNA samples were incubated for 2 minutes at 37 °C followed by heat-inactivation of the enzyme at 50 °C for 10 minutes. Samples were then directly processed for library preparation.

Additionally, deamination repair for 8 samples was also tested using the GeneRead DNA FFPE Kit (Ref 180134, Qiagen, Hilden Germany) as this kit includes an UDG incubation step during DNA isolation.

Supplementary Methods 3: Bioinformatics and Data Analysis

Data analysis was performed using Torrent Server™ v 5.6 and Ion Reporter™ Software v5.10 (Thermo Fisher Scientific, Waltham, MA, USA) for TMB analysis, filtering and annotations (the analysis was started with Ion Reporter v5.6 but after a server update all samples were reanalyzed using v5.10 as this provided improved bioinformatics for variant filtering and TMB calculation). The sequencing run was considered successful and the quality adequate when the following quality metrics were met: Mapped reads ≥ 300,000; average base coverage depth ≥ 300; amplicons with at least 300 reads ≥ 99%; absence of strand bias ≥ 95%; amplicons read end-to-end ≥ 99%. Integrative Genome Viewer (IGV) was utilized for visualization. The cut-off was set at 300× coverage and minimum 5% allelic frequency. Mutations with an allele frequency greater than 5% were considered for the calculation of TMB per standard protocol of the Oncomine TML panel.

Supplementary Methods 4: Immunohistochemistry for PD-L1 and CD8 Expression

Immunohistochemistry was performed on 3 µm tumor sections with a Ventana Benchmark Ultra (Ventana, Tucson, AZ, USA).

For CD8 staining, the CONFIRM anti-CD8 (SP57) Rabbit Monoclonal Primary Antibody (ref 790-4460, Ventana) was used. In brief, antigen retrieval was performed by EDTA retrieval for 64 minutes (protocol CC1) followed by 20 minutes of antibody incubation and staining using the UltraView DAB kit (ref 760-500).

For PD-L1 staining, the FDA-approved PD-L1 IHC 22C3 pharmDx assay (Dako Agilent, Santa Clara, USA) was used. Antigen retrieval was performed using the CC1 protocol and antibody incubation was performed for 32 minutes according to manufacturer’s instructions. Signal amplification was performed using the OptiView Amplifier kit (Ventana, ref 760-099) for 12 minutes followed by staining using the OptiView DAB IHC kit (Ventana, ref 760-700).

Analysis of IHC was performed by four board-certified senior pathologists (ELM, VH, MI and PH) independently and blinded of clinical outcome.

Supplementary Methods 5: Machine learning and mathematical modeling

Logistic regression and decision trees implemented in the Scikit-learn software were used to investigate combinations of biomarkers. The regularization penalty of the logistic regression was set to zero and the maximum depths of decision trees were set to the number of features (other parameters set to default values). Five-fold cross-validation repeated 30 times was applied to assess the prediction performance of combining biomarkers. The proportion of subjects with DCB in the whole lung or melanoma dataset was preserved in each fold. The mean and standard deviation of accuracy, precision, specificity and sensitivity were computed on all test sets.

Combination of biomarkers was performed only on samples where all the data of biomarkers was present: 24 samples in the NSCLC cohort and 22 samples in the melanoma cohort.


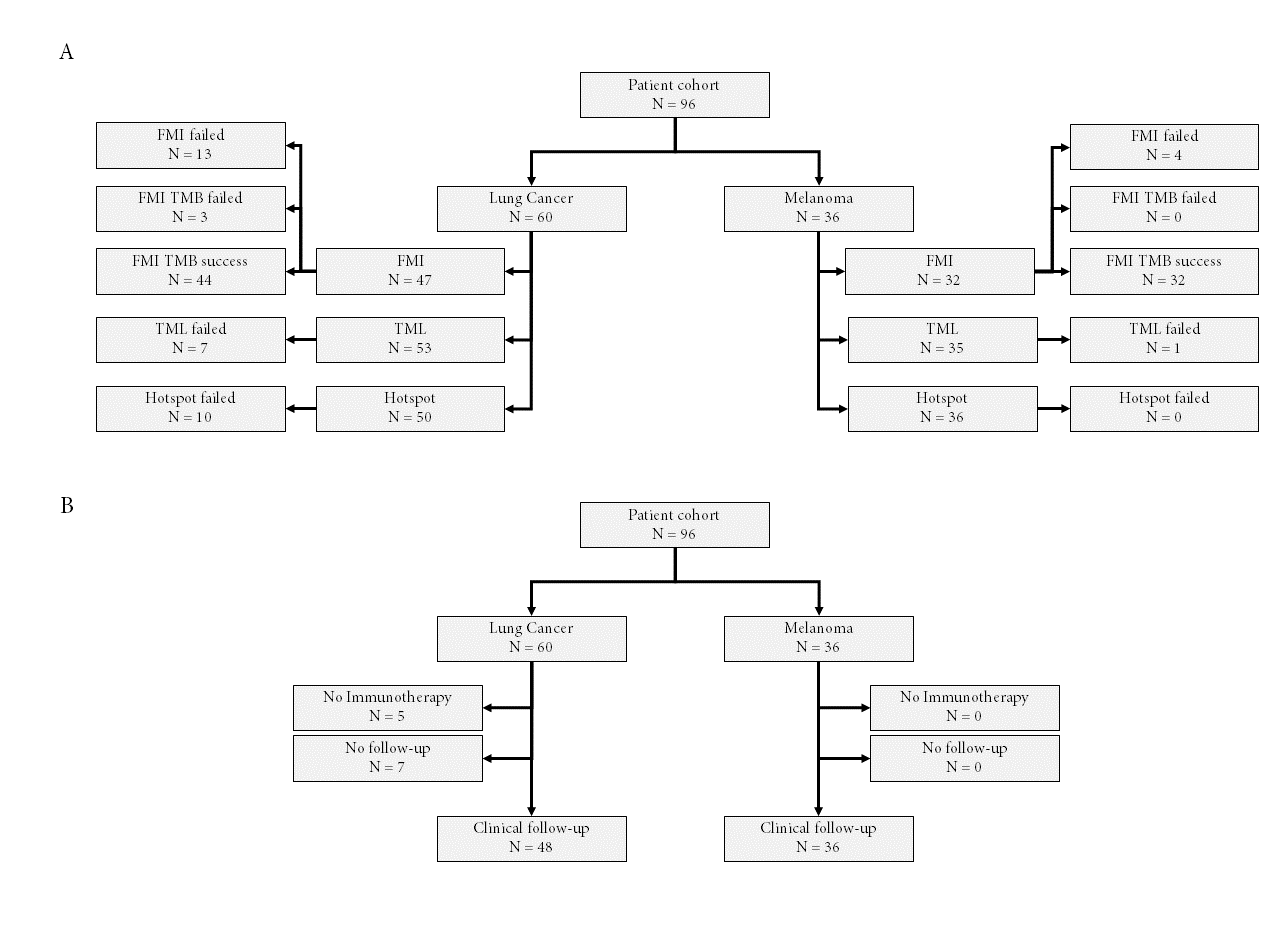


**Figure S1.** Flowchart of samples tested using the different sequencing panels. (**A**) Patient selection and (**B**) clinical follow-up allowing the calculation of durable clinical benefit (DCB). FMI = FoundationOne^®^, TML = Oncomine™ TML panel, Hotspot = Ion Ampliseq™ Hotspot V2 panel, N = number of patients. FMI TMB failed = A report with detected mutations was send out by Foundation Medicine but there was not enough confidence to provide a TMB value.


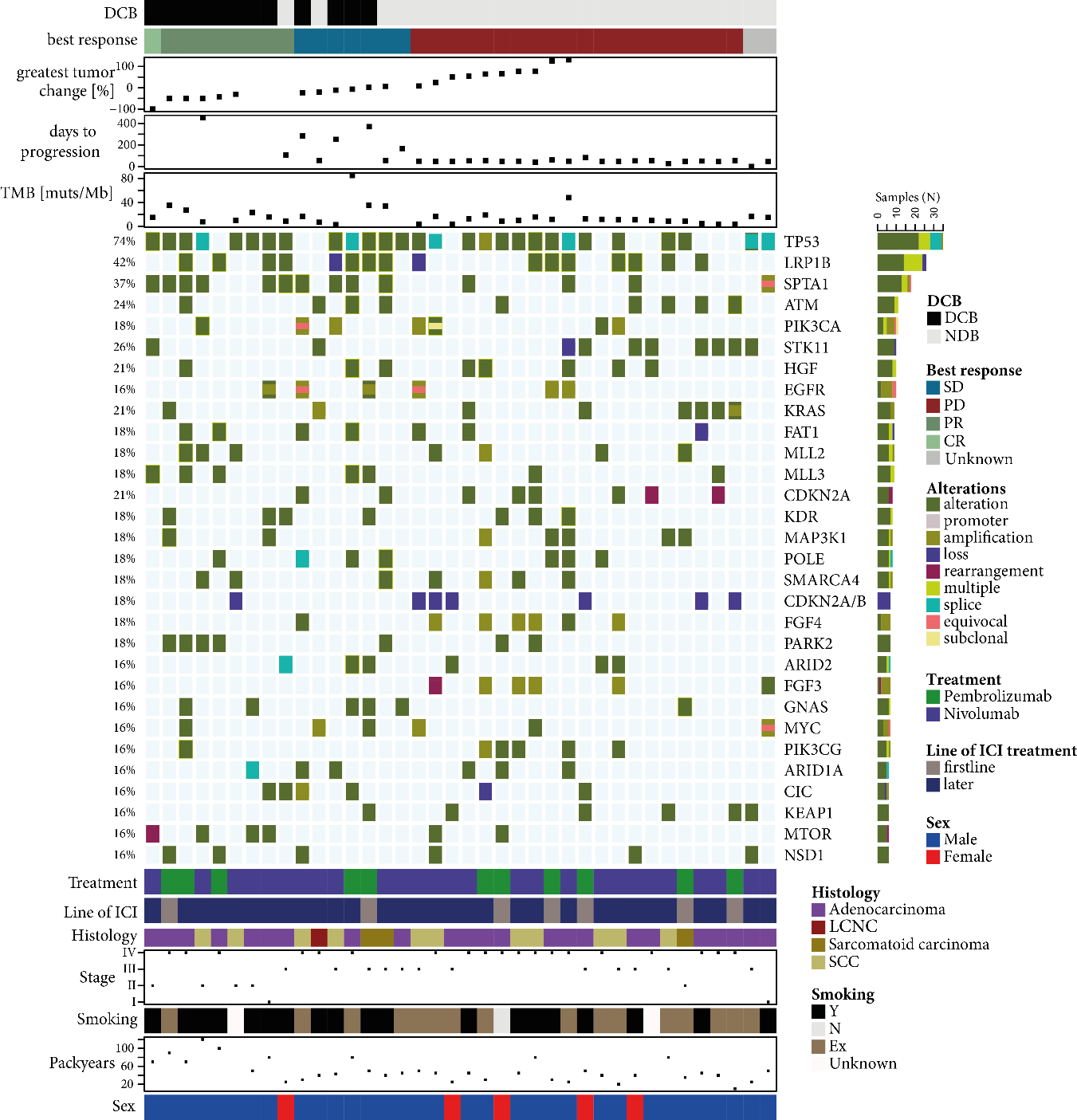


**Figure S2.** Oncoprint of genomic and clinical features of the NSCLC cohort as tested by FoundationOne^®^. Genetic alterations for the 30 genes with the highest mutation rate shown as detected by FoundationOne^®^ together with key clinical data. The samples are arranged by objective response and greatest baseline change. LCNC = large-cell neuroendocrine carcinoma, SCC = squamous cell carcinoma, Y = current smoker, N = never smoker, Ex = former smoker. Information on equivocal and subclonal mutation calls were retrieved from the report generated by Foundation Medicine.


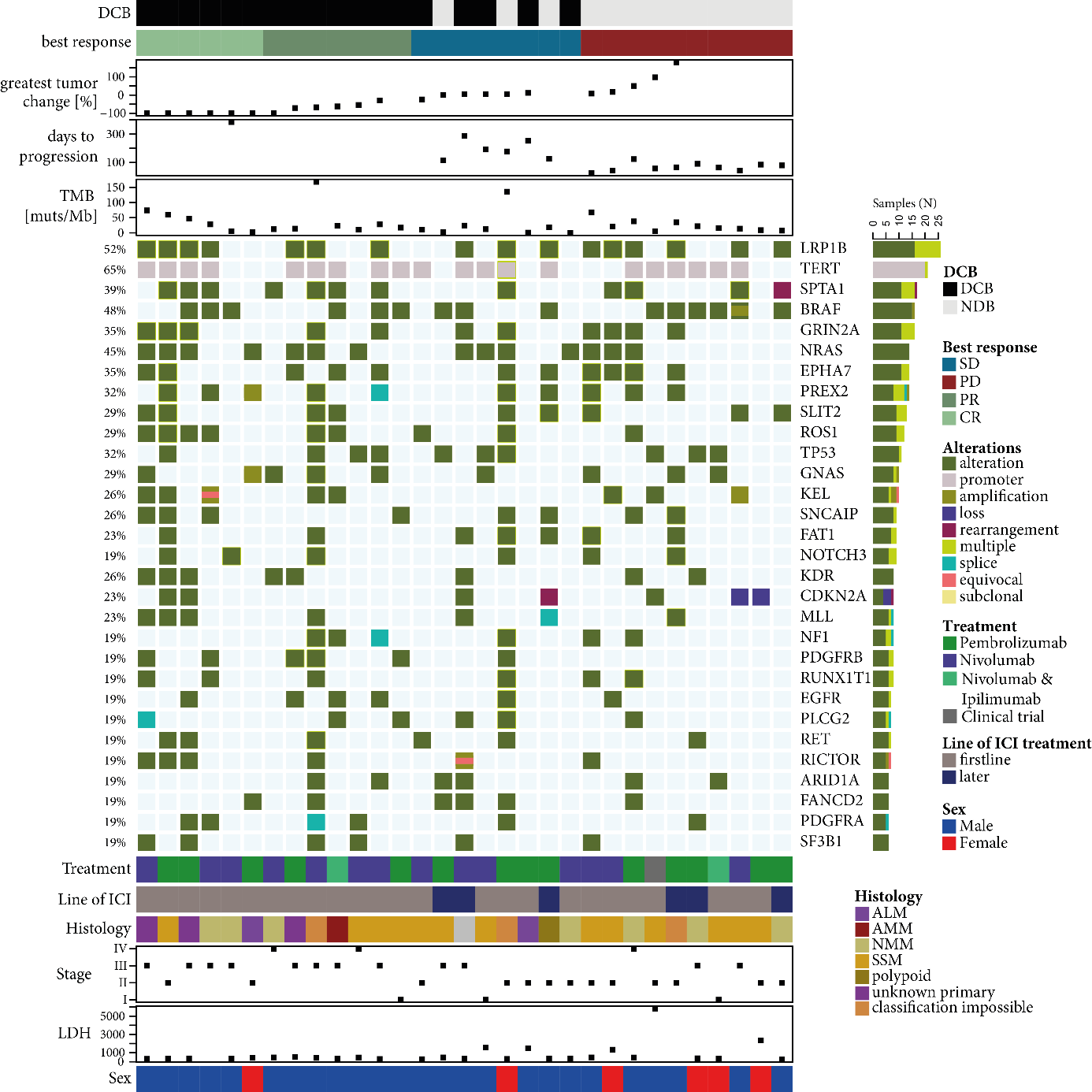


**Figure S3.** Oncoprint of genomic and clinical features of the melanoma cohort as tested by FoundationOne^®^. Genetic alterations for the 30 genes with the highest mutation rate as shown as detected by FoundationOne^®^ together with key clinical data. The samples are arranged by objective response and greatest baseline change. Abbreviations: ALM = acral lentiginous melanoma, AMM = amelanotic malignant melanoma, SSM = superficial spreading melanoma, NMM = nodular malignant melanoma. Information on equivocal and subclonal mutation calls were retrieved from the report generated by Foundation Medicine.


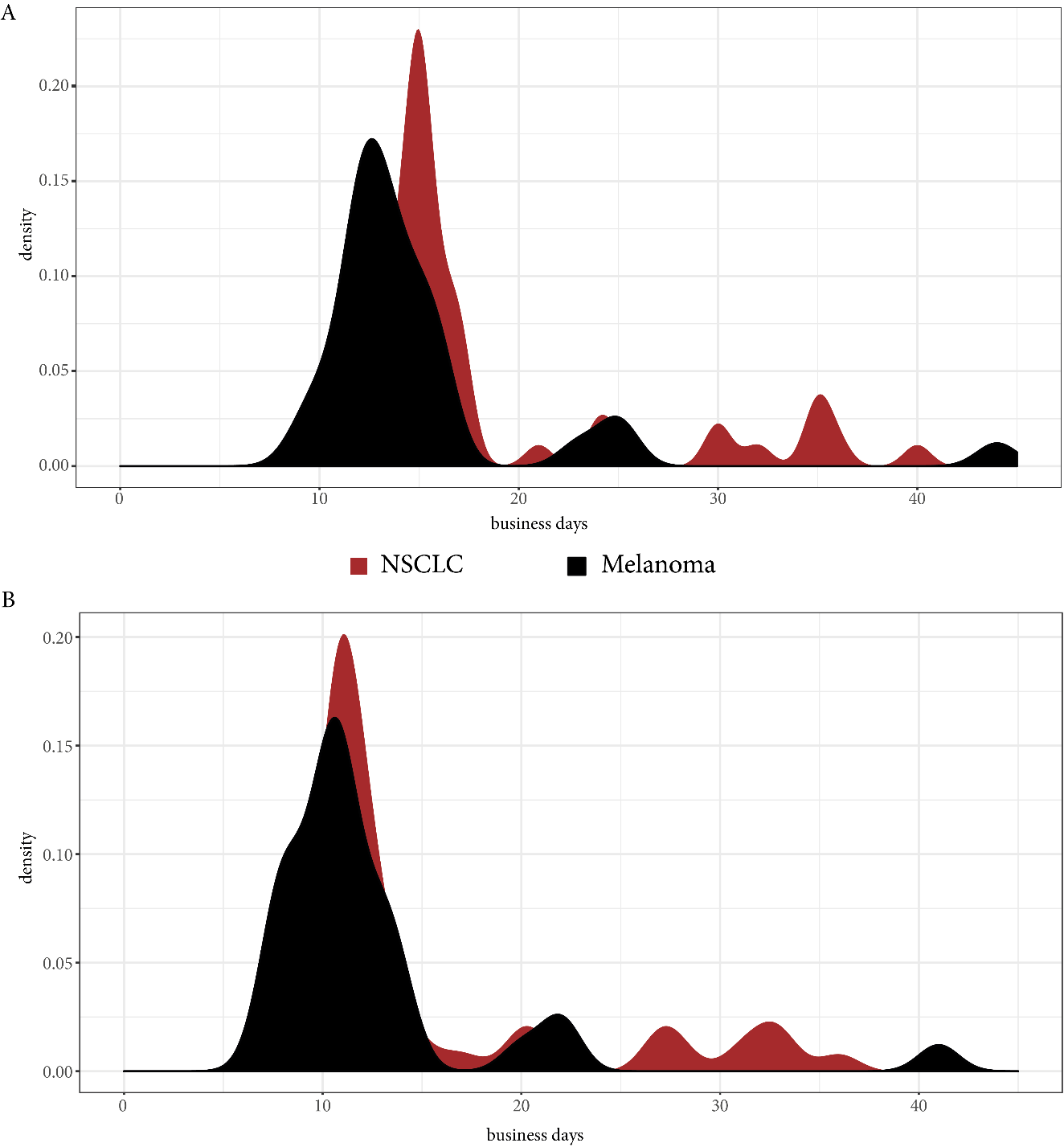


**Figure S4.** Turn-around time for samples sent to Foundation Medicine. (**A**) Turn-around time for sequencing in business days (Monday–Friday working week) from the date samples were sent to the date the final report was received (both days inclusive). (**B**) Turn-around time for sequencing time for FoundationOne^®^ in business days (from sample registration at Foundation Medicine until the final report, available as PDF).


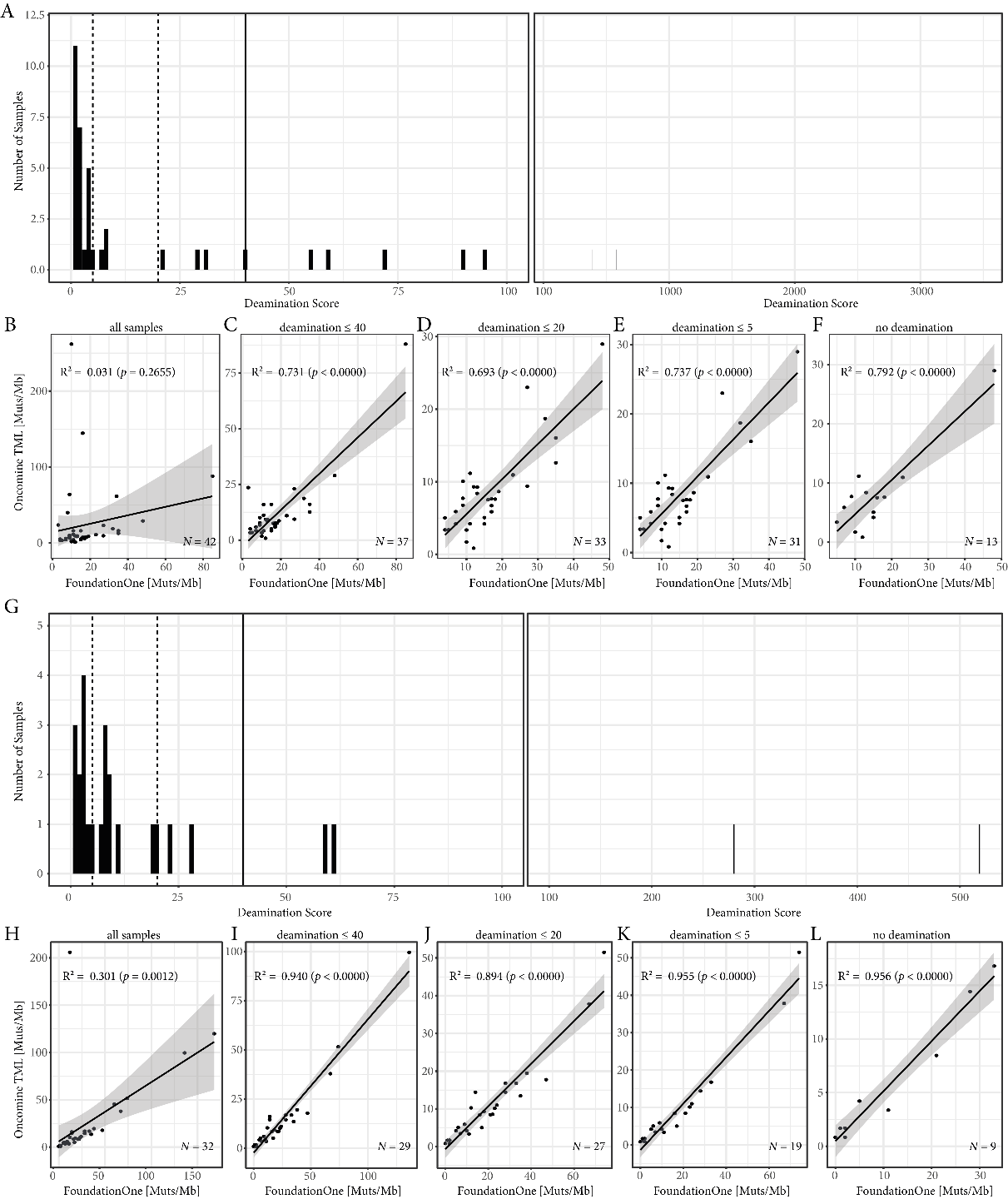


**Figure S5.** Deamination score of samples sequenced using the Oncomine™ TML panel. (**A**) Deamination scores obtained using the Oncomine™ TML panel in the NSCLC population. The numbers of samples with the respective deamination score are counted and are shown on the *y*-axis. The deamination scores at which correlation was computed are shown as a horizontal line. (**B**–**F**) Correlation of the TMB obtained from the Oncomine™ TML panel with the FoundationOne^®^ test for (**B**) all samples, (**C**) samples with a deamination score less than or equal than 40, (**D**) samples with a deamination score less than or equal to 20, (**E**) samples with a deamination score less than or equal to 5, and (**F**) for samples without any deamination (deamination score = 0). A deamination score of 40 was considered to be a reliable cut-off (**C**). (**G**) deamination score obtained using the Oncomine™ TML panel in the melanoma cohort. The number of samples with the respective deamination score are counted and are shown on the *y*-axis. The deamination scores at which correlation was computed are shown as a horizontal line. (**H**–**L**) correlation of the TMB obtained from the Oncomine™ TML panel with the FoundationOne^®^ test for (**H**) all samples, (**I**) samples with a deamination score less than or equal to 40, (**J**) samples with a deamination score less than or equal to 20, (**K**) samples with a deamination score less than or equal than 5, and (**L**) for samples without any deamination (deamination score = 0). Like in the NSCLC cohort, a deamination score of 40 was considered to be a reliable cut-off (**I**). *N* = number of samples used for analysis.


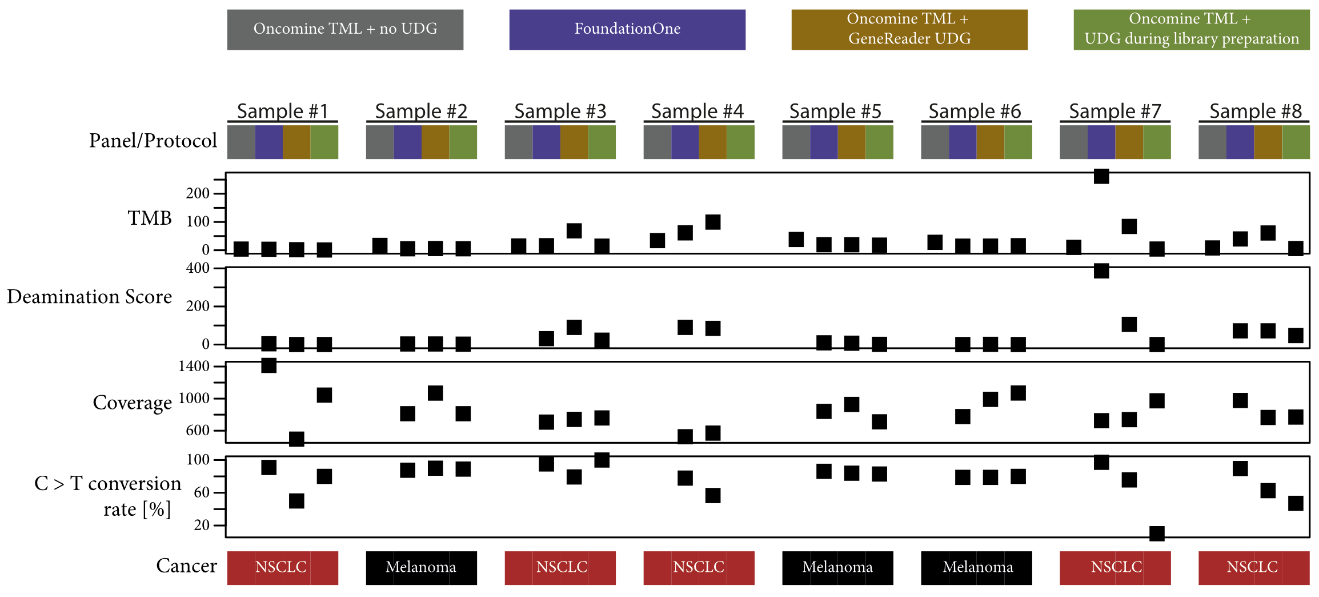


**Figure S6.** Impact of different deamination protocols on the C to T conversion rate (C > T) and the TMB using the Oncomine™ TML panel. To reduce deamination, UDG treatment was included in the sample preparation for subsequent sequencing. Deamination repair was either used during DNA extraction utilizing the Qiagen GeneReader™ DNA FFPE kit that includes a UDG treatment step during extraction or by directly adding UDG to the samples prior to library preparation as recommended by Thermo Fisher. In total, eight samples were tested using the respective protocols.


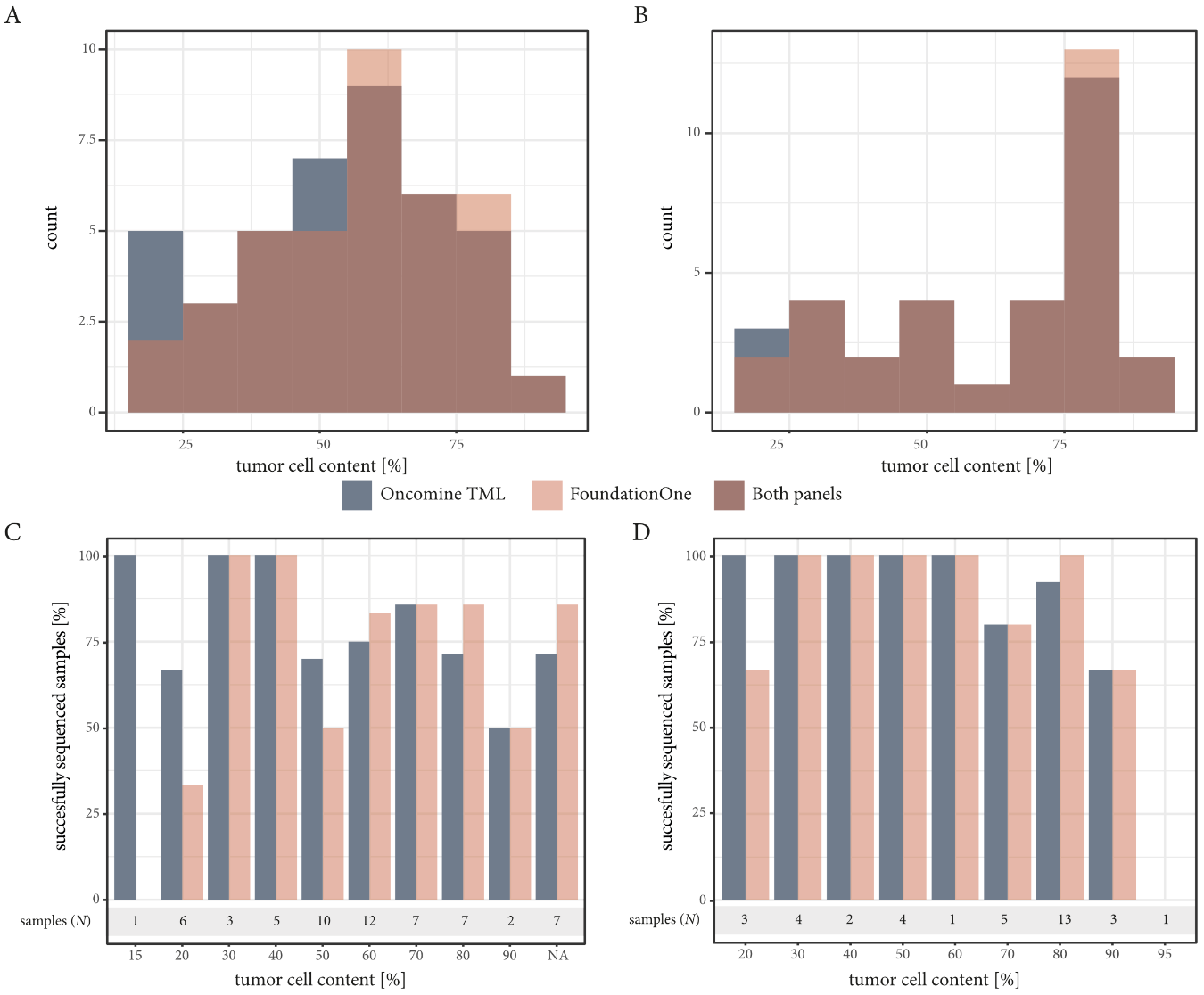


**Figure S7.** Influence of tumor cell content on sequencing results. Histograms of tumor cell ratio (in %) are shown for the successfully sequenced samples from (**A**) NSCLC and (**B**) melanoma patients. A higher proportion of samples with a lower tumor cell ratio was successfully sequenced using the Oncomine™ TML panel. Overlapping data of FoundationOne^®^ and Oncomine™ TML leads to a dark brown color. The proportion of successfully sequenced samples for each tumor cell ratio is shown for samples from (**C**) NSCLC and (**D**) melanoma patients. The number of samples (*N*) with the respective tumor cell ratio is shown below the graph.


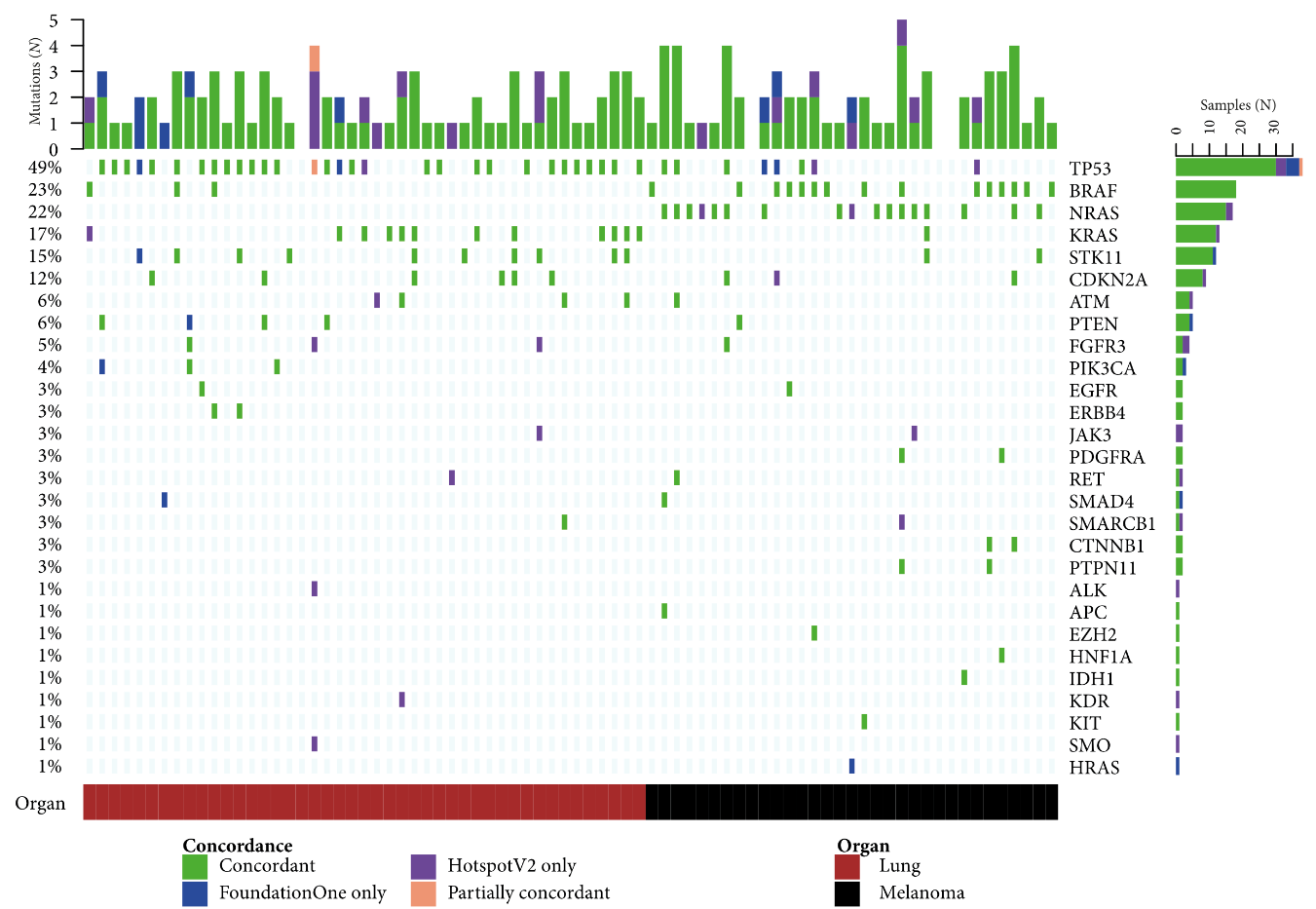


**Figure S8.** Comparison of sequencing results between FoundationOne^®^ and Hotspot V2. Only regions that are covered on the Hotspot V2 panel are compared. All mutations that were exclusively detected in the FoundationOne^®^ assay were controlled manually and were also present in the raw sequencing data of the Hotspot V2 test but filtered out due to an allele frequency < 5% or low amplicon quality. In partially concordant samples, multiple mutations per gene were detected but only some of them were detected in both tests.


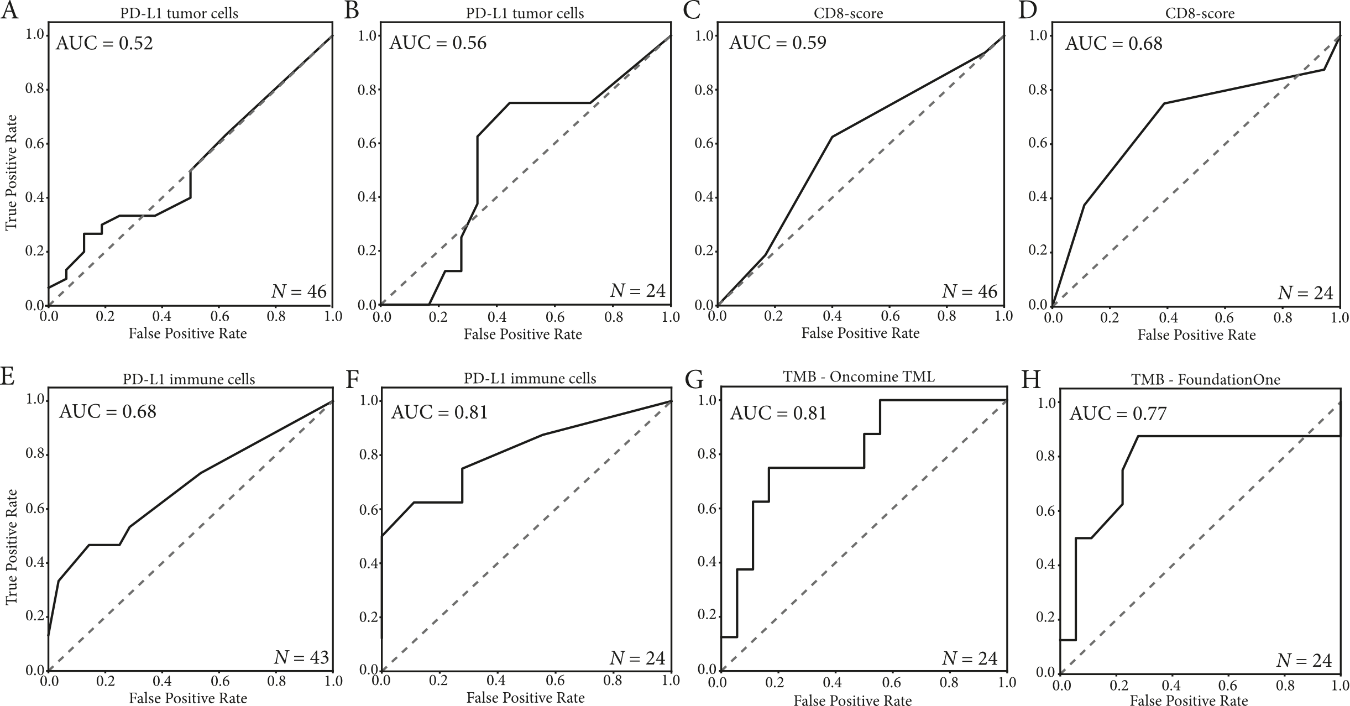


**Figure S9.** Receiver operator characteristics (ROC) curves calculated for the assessment of DCB in the NSCLC cohort. PD-L1 in tumor cells on the (**A**) whole dataset and on (**B**) the common dataset where all information for all the biomarkers was present (common dataset; *N* = 24) allowing the direct comparison of the respective biomarkers. (**C**) CD8-score from the whole and (**D**) the common dataset (*N* = 24). (E) PD-L1 expression in immune cells on the whole dataset and **(F**) the common dataset (*N* = 24). TMB on the common dataset obtained by (**G**) the oncomine TML panel and (**H**) the FoundationOne assay. Number of samples (*N*) is mentioned in the lower right corner of each figure.


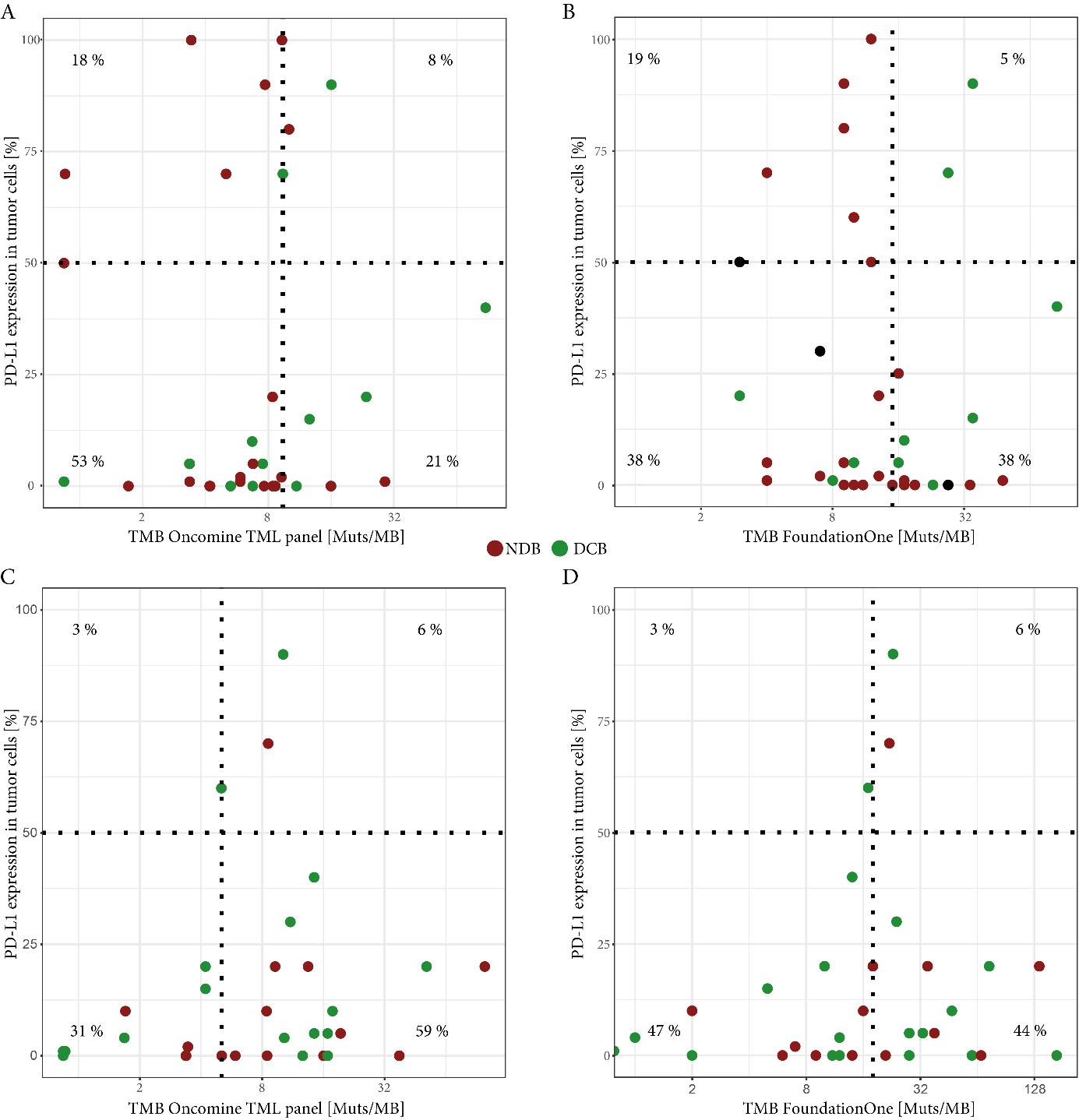


**Figure S10.** Correlation of PD-L1 expression in tumor cells and TMB with clinical outcome. (**A**) Tumor mutational burden using the OTML panel together with the PD-L1 expression in tumor cells is shown in NSCLC patients. (**B**) TMB assessed by FO together with PD-L1 is shown in NSCLC. (**C**) TMB using OTML and PD-L1 expression is shown in melanoma patients. (**D**) TMB by FO together with PD-L1 expression in tumor cells is shown in melanoma. The *x*-axis is log(2)-scaled for better representation of results.


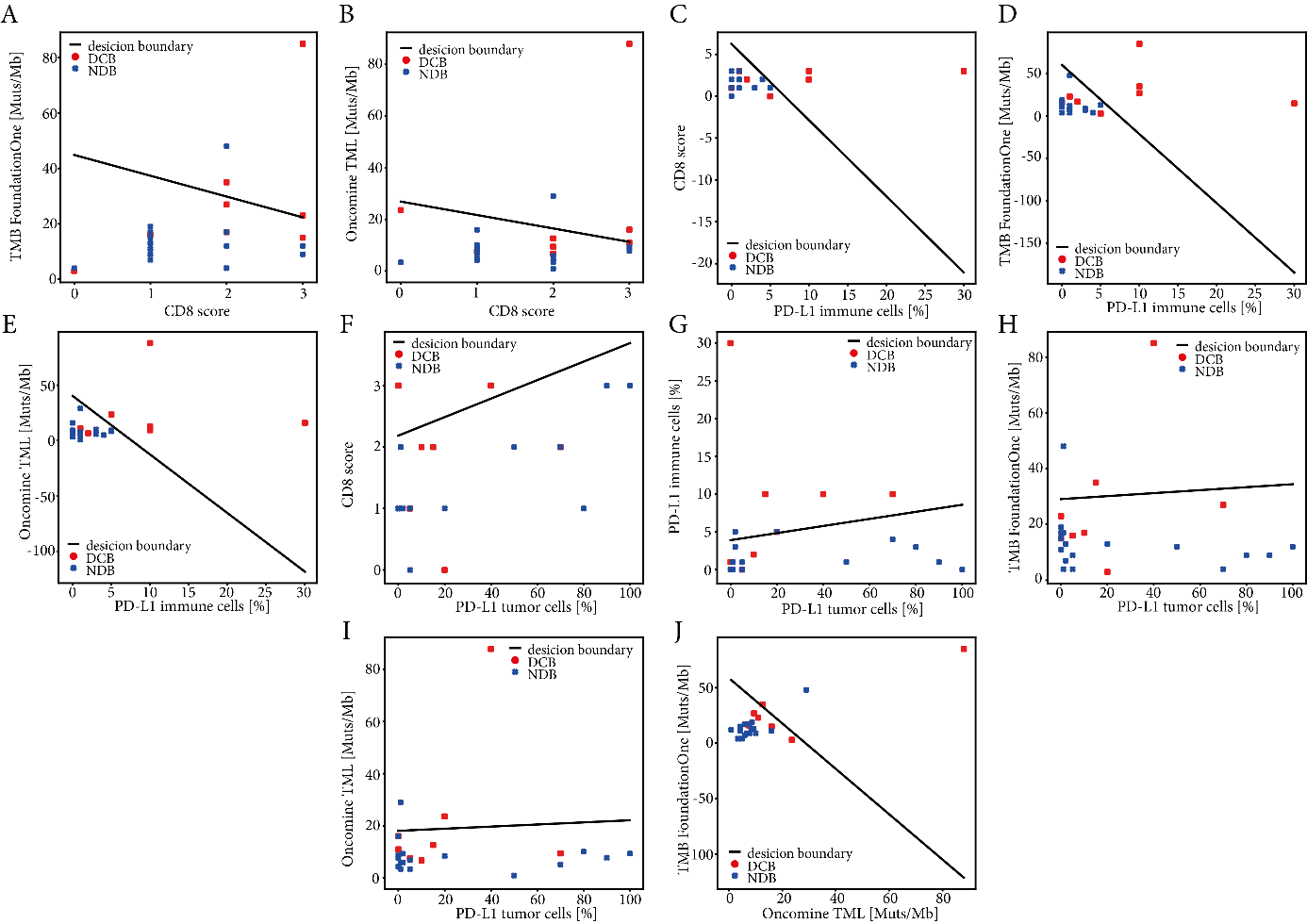


**Figure S11.** Logistic regression models built to predict durable clinical benefit (DCB) in the NSCLC cohort. Decision boundaries computed from the model are shown for each combination of the different datasets (filtered for samples where all the required data was present; *N* = 24); (**A**) TMB from FoundationOne^®^ combined with the CD8 score, (**B**) TMB derived from the Oncomine™ TML panel combined with CD8 score, (**C**) CD8 score combined with the PD-L1 expression in immune cells, (**D**) TMB obtained from FoundationOne^®^ combined with PD-L1 in immune cells, (**E**) TMB derived from the Oncomine™ TML panel combined with the PD-L1 expression in immune cells, (**F**) CD8 score combined with PD-L1 expression in immune cells, (**G**) PD-L1 expression in immune cells combined with PD-L1 expression in tumor cells, (**H**) TMB obtained from FoundationOne^®^ combined with PD-L1 expression in tumor cells, (**I**) TMB obtained from the Oncomine™ TML panel combined with PD-L1 expression in tumor cells, (**J**) TMB obtained from FoundationOne^®^ combined with TMB obtained by Oncomine™ TML.


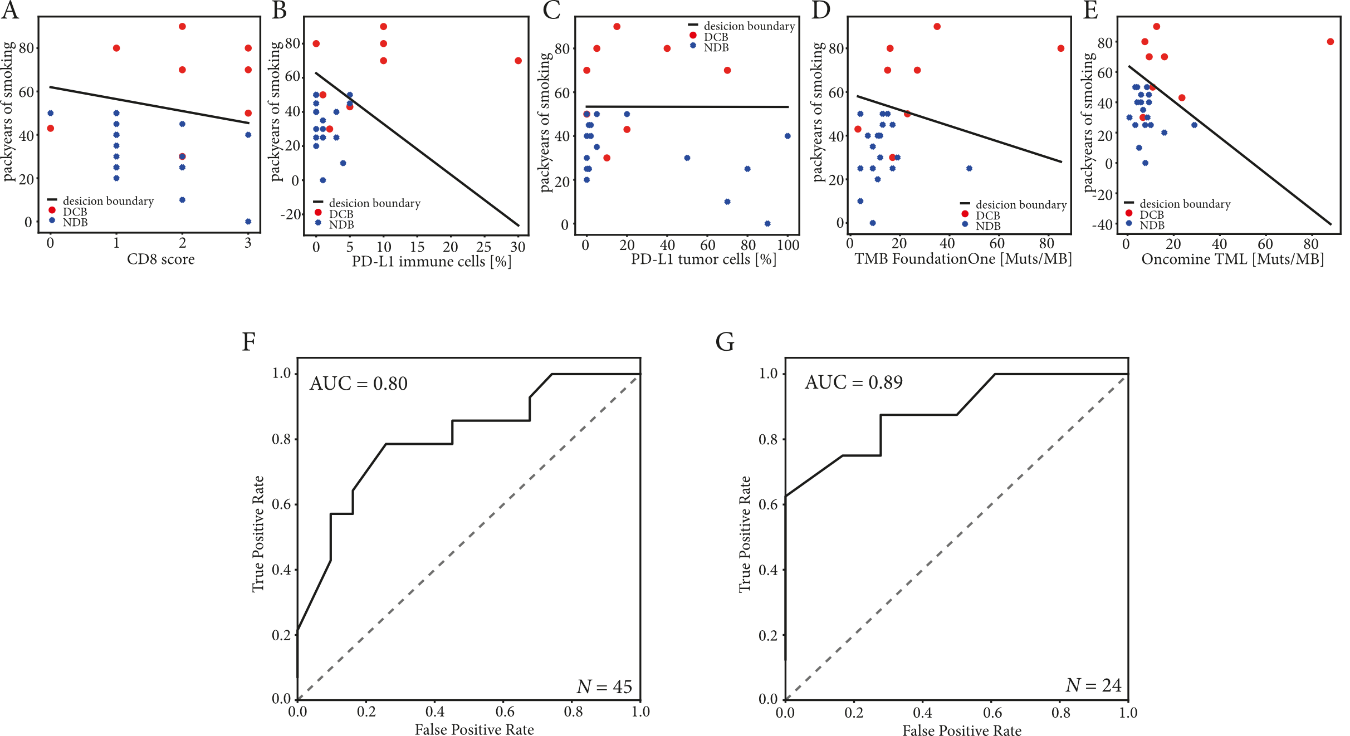


**Figure S12.** Packyears of smoking as biomarker. Influence of incorporation of packyears of smoking as biomarker in logistic regression models to predict durable clinical benefit in NSCLC patients (N = 24) together with (**A**) CD8-score, (**B**) PD-L1 expression in immune cells, (**C**) PD-L1 expression in tumor cells, (**D**) TMB assessed by FoundationOne^®^ and (**E**) TMB by Oncomine™ TML. Decision boundaries are shown with solid lines. The ROC of packyears of smoking alone is shown in (**F**) for all available data and in (**G**) for the common samples where data was present from both TMBs to allow comparison (*N* = 24). Number of samples (*N*) used is shown in the lower right corner of the figures.

**Table S1.** Characteristics of non-small cell lung cancer patients.

|  | **All Patients** | **DCB ^a^** | **NDB ^a^** | ***p*-value**  **(DCB ~ NDB)** |
| --- | --- | --- | --- | --- |
| ***N*** | 60 | 16 | 32 |  |
| Age |  |  |  | 0.3043 |
| Median (Range) | 63 (38–83) | 64 (46–83) | 61 (38–82) |  |
| Sex |  |  |  | 0.2395 |
| Female | 12 | 1 | 7 |  |
| Male | 48 | 15 | 25 |  |
| Smoking |  |  |  | 0.7359 |
| Active | 27 | 8 | 15 |  |
| Former | 27 | 7 | 14 |  |
| Never | 2 | 0 | 2 |  |
| Unknown | 4 | 1 | 1 |  |
| Packyears  Median (Range) |  | 70 (30–120) | 40 (10–90) | 0.0016 |
| Diagnosis |  |  |  | 0.9154 |
| Adenocarcinoma | 43 | 11 | 21 |  |
| Squamous Cell Carcinoma | 13 | 4 | 8 |  |
| Large Cell Neuroendocrine Carcinoma | 1 | 0 | 1 |  |
| Sarcomatoid Carcinoma | 3 | 1 | 2 |  |
| Stage |  |  |  | 0.0468 |
| I–II | 9 | 5 | 2 |  |
| III | 19 | 3 | 13 |  |
| IV | 32 | 8 | 17 |  |
| Treatment Line |  |  |  | 1.0000 |
| First-Line | 11 | 2 | 5 |  |
| Second-Line or Later | 41 | 14 | 27 |  |
| No ICI | 8 | 0 | 0 |  |
| Treatment Drug |  |  |  | 0.3100 |
| Nivolumab | 35 | 10 | 25 |  |
| Pembrolizumab | 17 | 6 | 7 |  |

^a^ The Clinical data was only available for 48 of the 60 samples.

**Table S2.** Characteristics of melanoma patients.

|  | **All patients** | **DCB** | **NDB** | ***p*-value**  **(DCB ~ NDB)** |
| --- | --- | --- | --- | --- |
| ***N*** | 36 | 22 | 14 |  |
| **Age** |  |  |  | 0.7425 |
| Median (Range) | 68 (26–86) | 69 (27–86) | 68 (26–82) |  |
| **Sex** |  |  |  | 0.4143 |
| Female | 7 | 2 | 5 |  |
| Male | 29 | 20 | 9 |  |
| **Diagnosis** |  |  |  | 0.3665 |
| ALM | 3 | 3 | 0 |  |
| AMM | 1 | 1 | 0 |  |
| SSM | 14 | 6 | 8 |  |
| NMM | 8 | 5 | 3 |  |
| Polypoid | 2 | 1 | 1 |  |
| Unknown Primary | 3 | 3 | 0 |  |
| Classification Impossible | 5 | 3 | 2 |  |
| **Stage** |  |  |  | 0.1210 |
| I–II | 18 | 8 | 10 |  |
| III | 14 | 11 | 3 |  |
| IV | 4 | 3 | 1 |  |
| **Treatment Line** |  |  |  | 0.1571 |
| First-Line | 30 | 21 | 9 |  |
| Second-Line or Later | 6 | 1 | 5 |  |
| **Treatment Drug** |  |  |  | 0.2413 |
| Nivolumab | 17 | 13 | 4 |  |
| Pembrolizumab | 16 | 8 | 8 |  |
| Nivolumab + Ipilimumab | 2 | 1 | 1 |  |
| Clinical trial (PDR001) | 1 | 0 | 1 |  |
| **LDH [U/l]** |  |  |  | 0.1352 |
| Mean (Range) | 388.5 (200 – 5832) | 370 (200 – 1567) | 474 (248 – 5832) |  |

Abbreviations: ALM = acral lentiginous melanoma, AMM = amelanotic malignant melanoma, SSM = superficial spreading melanoma, NMM = nodular malignant melanoma.

**Table S3.** Prediction of DCB from a single biomarker in non-small cell lung cancer patients.

| Biomarker | *N* | AUC | Optimal threshold | Threshold - Youden Index | Sensitivity ^a^ | Specificity ^a^ |
| --- | --- | --- | --- | --- | --- | --- |
| Packyears Smoking | 45 | 0.89 | 50 | 70 | 0.75 | 0.83 |
| PD-L1 in Tumor Cells | 46 | 0.56 | 10 | 5 | 0.63 | 0.67 |
| PD-L1 in Immune Cells | 43 | 0.81 | 2 | 5 | 0.75 | 0.72 |
| CD8 Score | 46 | 0.68 | 2 | 2 | 0.75 | 0.61 |
| TMB FO | 36 | 0.77 | 15 | 15 | 0.88 | 0.72 |
| TMB OM | 36 | 0.81 | 9.39 | 9.39 | 0.75 | 0.83 |

Abbreviations: FO = FoundationOne^®^, OM = Oncomine™ TML, ^a^ the sensitivity and specificity is calculated for the threshold using the optimal threshold (minimal distance to (0, 1) corner). *N* = Number of samples used for calculation.

**Table S4.** Prediction of DCB from a single biomarker in melanoma patients.

| Biomarker | *N* | AUC | Optimal threshold | Threshold - Youden Index | Sensitivity ^a^ | Specificity ^a^ |
| --- | --- | --- | --- | --- | --- | --- |
| PD-L1 in Tumor Cells | 36 | 0.53 | 4 | 1 | 0.67 | 0.46 |
| PD-L1 in Immune Cells | 32 | 0.53^b^ | 5 | 5 | 0.50 | 0.62 |
| CD8 Score | 36 | 0.59 | 3 | 3 | 0.42 | 0.77 |
| TMB FO | 32 | 0.55^b^ | 18 | 6 | 0.58 | 0.54 |
| TMB OM | 32 | 0.53^b^ | 5.06 | 5.06 | 0.42 | 0.85 |

Abbreviations: FO = FoundationOne^®^, OM = Oncomine™ TML, ^a^ the sensitivity and specificity is calculated for the threshold using the optimal threshold (minimal distance to (0, 1) corner). ^b^ The ROC favors the “low” population and not the “high” population. *N* = Number of samples used for calculation.

**Table S5.** Prediction of durable clinical benefit in non-small cell lung cancer using single biomarkers.

|  |  | AUC | | Precision | | Accuracy | | Sensitivity | | Specificity | |
| --- | --- | --- | --- | --- | --- | --- | --- | --- | --- | --- | --- |
| **Biomarker** | ***N*** | **Mean** | **SD** | **Mean** | **SD** | **Mean** | **SD** | **Mean** | **SD** | **Mean** | **SD** |
| TMB OM | 36 | 0.70 | 0.23 | 0.56 | 0.40 | 0.76 | 0.18 | 0.57 | 0.41 | 0.84 | 0.19 |
| TMB FO | 36 | 0.69 | 0.19 | 0.47 | 0.34 | 0.70 | 0.17 | 0.62 | 0.40 | 0.74 | 0.23 |
| PD-L1 Tumor | 46 | 0.46 | 0.20 | 0.01 | 0.04 | 0.67 | 0.12 | 0.02 | 0.11 | 0.95 | 0.19 |
| PD-L1 Immune | 43 | 0.73 | 0.21 | 0.63 | 0.47 | 0.83 | 0.14 | 0.50 | 0.41 | 0.97 | 0.15 |
| CD8 Score | 46 | 0.54 | 0.20 | 0.04 | 0.11 | 0.62 | 0.15 | 0.06 | 0.19 | 0.85 | 0.24 |

Abbreviations: FO = FoundationOne^®^, OM = Oncomine™ TML, *N* = Number of samples used for calculation.

**Table S6.** Prediction of durable clinical benefit in non-small cell lung cancer patients by combining two biomarkers using decision trees (*N* = 24).

|  | AUC | | Precision | | Accuracy | | Sensitivity | | Specificity | |
| --- | --- | --- | --- | --- | --- | --- | --- | --- | --- | --- |
| **Biomarker** | **Mean** | **SD** | **Mean** | **SD** | **Mean** | **SD** | **Mean** | **SD** | **Mean** | **SD** |
| PD-L1 Tumor and CD8 Score | 0.55 | 0.25 | 0.11 | 0.25 | 0.58 | 0.15 | 0.13 | 0.27 | 0.78 | 0.22 |
| PD-L1 Immune and CD8 Score | 0.64 | 0.28 | 0.62 | 0.46 | 0.82 | 0.13 | 0.50 | 0.41 | 0.96 | 0.11 |
| PD-L1 Tumor and PD-L1 Immune | 0.64 | 0.29 | 0.61 | 0.46 | 0.82 | 0.13 | 0.49 | 0.41 | 0.96 | 0.13 |
| PD-L1 Tumor and TMB OM | 0.66 | 0.27 | 0.30 | 0.37 | 0.68 | 0.15 | 0.34 | 0.41 | 0.83 | 0.19 |
| CD8 Score and TMB OM | 0.67 | 0.26 | 0.31 | 0.41 | 0.69 | 0.17 | 0.33 | 0.42 | 0.85 | 0.20 |
| PD-L1 Immune and TMB OM | 0.68 | 0.28 | 0.54 | 0.46 | 0.78 | 0.16 | 0.45 | 0.40 | 0.92 | 0.18 |
| PD-L1 Immune and TMB FO | 0.71 | 0.25 | 0.58 | 0.47 | 0.80 | 0.14 | 0.44 | 0.40 | 0.96 | 0.14 |
| TMB OM and TMB FO | 0.71 | 0.29 | 0.28 | 0.41 | 0.70 | 0.16 | 0.26 | 0.37 | 0.90 | 0.18 |
| CD8 Score and TMB FO | 0.72 | 0.23 | 0.38 | 0.42 | 0.72 | 0.16 | 0.38 | 0.41 | 0.86 | 0.21 |
| PD-L1 Tumor and TMB FO | 0.73 | 0.26 | 0.59 | 0.48 | 0.80 | 0.15 | 0.45 | 0.40 | 0.96 | 0.12 |

Abbreviations: FO = FoundationOne^®^, OM = Oncomine™ TML.

**Table S7.** Prediction of durable clinical benefit in non-small cell lung cancer patients by combining two biomarkers using logistic regression models (*N* = 24).

|  | AUC | | Precision | | Accuracy | | Sensitivity | | Specificity | |
| --- | --- | --- | --- | --- | --- | --- | --- | --- | --- | --- |
| **Biomarker** | **Mean** | **SD** | **Mean** | **SD** | **Mean** | **SD** | **Mean** | **SD** | **Mean** | **SD** |
| PD-L1 Tumor and CD8 Score | 0.59 | 0.32 | 0.29 | 0.40 | 0.69 | 0.15 | 0.26 | 0.35 | 0.88 | 0.20 |
| PD-L1 Tumor and TMB FO | 0.66 | 0.30 | 0.28 | 0.41 | 0.72 | 0.14 | 0.26 | 0.38 | 0.93 | 0.15 |
| PD-L1 Tumor and TMB OM | 0.71 | 0.25 | 0.25 | 0.39 | 0.71 | 0.13 | 0.23 | 0.36 | 0.91 | 0.17 |
| PD-L1 Immune and CD8 Score | 0.72 | 0.29 | 0.57 | 0.44 | 0.78 | 0.16 | 0.50 | 0.41 | 0.90 | 0.17 |
| CD8 Score and TMB FO | 0.73 | 0.29 | 0.28 | 0.41 | 0.70 | 0.16 | 0.26 | 0.37 | 0.90 | 0.18 |
| CD8 Score and TMB OM | 0.73 | 0.29 | 0.28 | 0.41 | 0.70 | 0.16 | 0.26 | 0.37 | 0.90 | 0.18 |
| TMB OM and TMB FO | 0.73 | 0.29 | 0.22 | 0.37 | 0.71 | 0.13 | 0.22 | 0.37 | 0.92 | 0.17 |
| PD-L1 Immune and TMB OM | 0.80 | 0.24 | 0.67 | 0.42 | 0.81 | 0.17 | 0.59 | 0.40 | 0.91 | 0.18 |
| PD-L1 Immune and TMB FO | 0.81 | 0.23 | 0.56 | 0.43 | 0.76 | 0.17 | 0.52 | 0.41 | 0.87 | 0.20 |
| PD-L1 Tumor and PD-L1 Immune | 0.83 | 0.24 | 0.56 | 0.42 | 0.78 | 0.15 | 0.54 | 0.41 | 0.88 | 0.19 |

Abbreviations: FO = FoundationOne^®^, OM = Oncomine™ TML.

**Table S8.** Integrating packyears of smoking as biomarker for the prediction of DCB in non-small cell lung cancer patients using logistic regression models (*N* = 24).

|  | AUC | | Precision | | Accuracy | | Sensitivity | | Specificity | |
| --- | --- | --- | --- | --- | --- | --- | --- | --- | --- | --- |
| **Biomarker** | **Mean** | **SD** | **Mean** | **SD** | **Mean** | **SD** | **Mean** | **SD** | **Mean** | **SD** |
| PY | 0.80 | 0.21 | 0.76 | 0.42 | 0.87 | 0.15 | 0.62 | 0.40 | 0.97 | 0.15 |
| CD8 Score and PY | 0.85 | 0.26 | 0.75 | 0.40 | 0.87 | 0.15 | 0.67 | 0.39 | 0.95 | 0.13 |
| TMB FO and PY | 0.87 | 0.24 | 0.71 | 0.41 | 0.85 | 0.14 | 0.63 | 0.39 | 0.94 | 0.13 |
| PD-L1 Tumor and PY | 0.88 | 0.20 | 0.69 | 0.42 | 0.83 | 0.15 | 0.62 | 0.40 | 0.92 | 0.16 |
| PD-L1 Immune and PY | 0.89 | 0.19 | 0.61 | 0.41 | 0.81 | 0.15 | 0.60 | 0.41 | 0.89 | 0.17 |
| TMB OM and PY | 0.89 | 0.21 | 0.77 | 0.36 | 0.87 | 0.14 | 0.71 | 0.36 | 0.93 | 0.13 |

Abbreviations: FO = FoundationOne^®^, OM = Oncomine™ TML, PY = Packyears of smoking.

**Table S9.** Prediction of durable clinical benefit in melanoma patients using single biomarkers.

|  |  | AUC | | Precision | | Accuracy | | Sensitivity | | Specificity | |
| --- | --- | --- | --- | --- | --- | --- | --- | --- | --- | --- | --- |
| **Biomarker** | ***N*** | **Mean** | **SD** | **Mean** | **SD** | **Mean** | **SD** | **Mean** | **SD** | **Mean** | **SD** |
| TMB OM | 32 | 0.43 | 0.15 | 0.22 | 0.31 | 0.44 | 0.16 | 0.19 | 0.26 | 0.67 | 0.35 |
| TMB FO | 32 | 0.36 | 0.15 | 0.12 | 0.18 | 0.36 | 0.16 | 0.15 | 0.23 | 0.56 | 0.40 |
| PD-L1 Tumor | 36 | 0.36 | 0.15 | 0.09 | 0.15 | 0.37 | 0.15 | 0.16 | 0.30 | 0.60 | 0.43 |
| PD-L1 Immune | 32 | 0.38 | 0.13 | 0.09 | 0.17 | 0.38 | 0.13 | 0.15 | 0.31 | 0.62 | 0.35 |
| CD8 Score | 36 | 0.49 | 0.19 | 0.38 | 0.35 | 0.49 | 0.17 | 0.33 | 0.29 | 0.63 | 0.30 |

Abbreviations: FO = FoundationOne^®^, OM = Oncomine™ TML, *N* = Number of samples used for calculation.

**Table S10.** Prediction of durable clinical benefit in melanoma patients by combining two biomarkers using decision trees (*N* = 22).

|  | AUC | | Precision | | Accuracy | | Sensitivity | | Specificity | |
| --- | --- | --- | --- | --- | --- | --- | --- | --- | --- | --- |
| **Biomarker** | **Mean** | **SD** | **Mean** | **SD** | **Mean** | **SD** | **Mean** | **SD** | **Mean** | **SD** |
| PD-L1 Tumor and CD8 Score | 0.31 | 0.17 | 0.20 | 0.26 | 0.40 | 0.15 | 0.19 | 0.24 | 0.59 | 0.30 |
| PD-L1 Immune and CD8 Score | 0.31 | 0.19 | 0.19 | 0.22 | 0.36 | 0.16 | 0.22 | 0.27 | 0.49 | 0.30 |
| PD-L1 Tumor and PD-L1 Immune | 0.26 | 0.18 | 0.15 | 0.22 | 0.30 | 0.16 | 0.18 | 0.24 | 0.43 | 0.33 |
| PD-L1 Tumor and TMB OM | 0.44 | 0.24 | 0.42 | 0.26 | 0.48 | 0.21 | 0.57 | 0.37 | 0.40 | 0.30 |
| CD8 Score and TMB OM | 0.46 | 0.24 | 0.41 | 0.27 | 0.49 | 0.19 | 0.54 | 0.36 | 0.45 | 0.32 |
| PD-L1 Immune and TMB OM | 0.45 | 0.22 | 0.41 | 0.29 | 0.48 | 0.20 | 0.51 | 0.37 | 0.46 | 0.32 |
| PD-L1 Immune and TMB FO | 0.30 | 0.16 | 0.18 | 0.23 | 0.35 | 0.15 | 0.23 | 0.31 | 0.48 | 0.33 |
| TMB OM and TMB FO | 0.46 | 0.25 | 0.42 | 0.27 | 0.49 | 0.21 | 0.56 | 0.37 | 0.44 | 0.33 |
| CD8 Score and TMB FO | 0.31 | 0.16 | 0.14 | 0.20 | 0.37 | 0.15 | 0.17 | 0.25 | 0.56 | 0.36 |
| PD-L1 Tumor and TMB FO | 0.30 | 0.18 | 0.11 | 0.19 | 0.34 | 0.16 | 0.13 | 0.24 | 0.54 | 0.37 |

Abbreviations: FO = FoundationOne^®^, OM = Oncomine™ TML.

**Table S11.** Prediction of durable clinical benefit in melanoma patients by combining two biomarkers using logistic regression models (*N* = 22).

|  | AUC | | Precision | | Accuracy | | Sensitivity | | Specificity | |
| --- | --- | --- | --- | --- | --- | --- | --- | --- | --- | --- |
| **Biomarker** | **Mean** | **SD** | **Mean** | **SD** | **Mean** | **SD** | **Mean** | **SD** | **Mean** | **SD** |
| PD-L1 Tumor and CD8 Score | 0.37 | 0.21 | 0.34 | 0.31 | 0.43 | 0.17 | 0.31 | 0.26 | 0.56 | 0.30 |
| PD-L1 Tumor and TMB FO | 0.33 | 0.26 | 0.26 | 0.28 | 0.35 | 0.18 | 0.28 | 0.26 | 0.42 | 0.36 |
| PD-L1 Tumor and TMB OM | 0.34 | 0.25 | 0.24 | 0.27 | 0.34 | 0.19 | 0.27 | 0.27 | 0.41 | 0.33 |
| PD-L1 Immune and CD8 Score | 0.38 | 0.25 | 0.28 | 0.30 | 0.41 | 0.18 | 0.26 | 0.26 | 0.54 | 0.33 |
| CD8 Score and TMB FO | 0.50 | 0.27 | 0.51 | 0.30 | 0.53 | 0.19 | 0.53 | 0.31 | 0.54 | 0.33 |
| CD8 Score and TMB OM | 0.49 | 0.25 | 0.45 | 0.30 | 0.48 | 0.17 | 0.47 | 0.32 | 0.50 | 0.33 |
| TMB OM and TMB FO | 0.33 | 0.25 | 0.31 | 0.26 | 0.34 | 0.20 | 0.37 | 0.31 | 0.31 | 0.31 |
| PD-L1 Immune and TMB OM | 0.34 | 0.22 | 0.29 | 0.25 | 0.37 | 0.16 | 0.35 | 0.31 | 0.40 | 0.30 |
| PD-L1 Immune and TMB FO | 0.36 | 0.24 | 0.32 | 0.27 | 0.40 | 0.17 | 0.36 | 0.30 | 0.45 | 0.33 |
| PD-L1 Tumor and PD-L1 Immune | 0.25 | 0.19 | 0.14 | 0.22 | 0.34 | 0.17 | 0.15 | 0.23 | 0.53 | 0.35 |

Abbreviations: FO = FoundationOne^®^, OM = Oncomine™ TML.

**Table S12.** Tables containing data of the NSCLC and Melanoma cohorts.

See the Excel table.

| 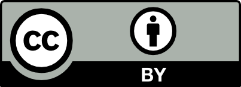 | © 2019 by the authors. Licensee MDPI, Basel, Switzerland. This article is an open access article distributed under the terms and conditions of the Creative Commons Attribution (CC BY) license (http://creativecommons.org/licenses/by/4.0/). |
| --- | --- |
